# Pemphigus Vulgaris Treated With Ibrutinib: A Case Report

**DOI:** 10.7759/cureus.61317

**Published:** 2024-05-29

**Authors:** Abdulellah I Aleissa, Hadeel F Alsulaimani, Tarek Mohamed

**Affiliations:** 1 Medicine, King Abdulaziz University Faculty of Medicine, Jeddah, SAU; 2 Dermatology, King Abdulaziz University Faculty of Medicine, Jeddah, SAU; 3 Hematology and Oncology, Dr. Soliman Fakeeh Hospital, Jeddah, SAU

**Keywords:** ibrutinib therapy, medical dermatology, bollous autoimmune diseases, pemphigus vulgaris, biological treatment

## Abstract

Pemphigus vulgaris (PV) stands as a rare autoimmune disorder characterized by blistering and erosion of mucocutaneous membranes. The pathogenesis of PV implicates both B and T cells, which target cell-to-cell adhesion molecules within the epithelia of the skin and oral mucosa, leading to acantholysis. Typically, the presentation involves blistering of the oral mucosa, often followed by cutaneous lesions. Given the considerable risk of morbidity and mortality associated with PV, early diagnosis is crucial, typically relying on a combination of clinical features, histopathology, and direct immunofluorescence. Bruton tyrosine kinase (BTK) plays a significant role in the pathophysiology of autoimmune diseases and inflammation. Herein, we present a case of PV that demonstrated resistance to first-line therapy with steroids. Subsequently, treatment with the BTK inhibitor ibrutinib was initiated, yielding favorable outcomes. This case underscores the potential of targeted therapies, such as BTK inhibitors, in managing PV refractory to conventional treatment modalities.

## Introduction

Pemphigus vulgaris (PV) is an autoimmune disorder characterized by the destruction of desmogleins, integral components of desmosomes responsible for cell adhesion [1]. Specifically, IgG antibodies target desmogleins 1 and 3, compromising their function and leading to erosion of the stratified squamous epithelia of the skin and oral mucosa [2]. Manifestations of PV typically include blisters and bullae affecting mucocutaneous membranes such as the oropharyngeal mucous membrane, anus, cervix, vagina, vocal cords, and eyes [3]. The median age of diagnosis in southern Saudi Arabia is reported to be 40 years [4], underscoring the importance of early recognition and intervention for improved prognosis. Diagnosis of PV relies on clinical presentation and the detection of autoantibodies via direct immunofluorescence microscopy [2]. Treatment strategies often involve corticosteroids and non-steroidal drugs, including immunosuppressive agents and immunoglobulins [3]. Notably, Bruton tyrosine kinase (BTK) inhibitors, which modulate signaling pathways in various cells, including B and T cells, have emerged as therapeutic options for PV [5]. Herein, we present the case of a male patient initially misdiagnosed with herpes zoster, who experienced significant symptomatic improvement following treatment with ibrutinib, highlighting the potential efficacy of targeted therapies in managing PV.

## Case presentation

A 35-year-old male presented to the hospital reporting painful mouth lesions that had emerged two weeks prior to consultation. The lesions initially manifested as bullae, subsequently leading to erosions, crust formation, and occasional bloody discharge, significantly impeding eating. Incidentally, the patient was diagnosed with stage 2 chronic lymphocytic leukemia (CLL) (ICD-10 code: C91.1) during peripheral blood investigations, despite the absence of constitutional symptoms or lymphadenopathy. Notably, the patient had undergone tonsillectomy two weeks prior to symptom onset. Initially misdiagnosed as lesions attributable to the herpes zoster virus, the patient was referred to another hospital for treatment. Despite receiving antiviral therapy for a duration of two weeks, there was no improvement observed, and the lesions continued to progress.

**Figure 1:**
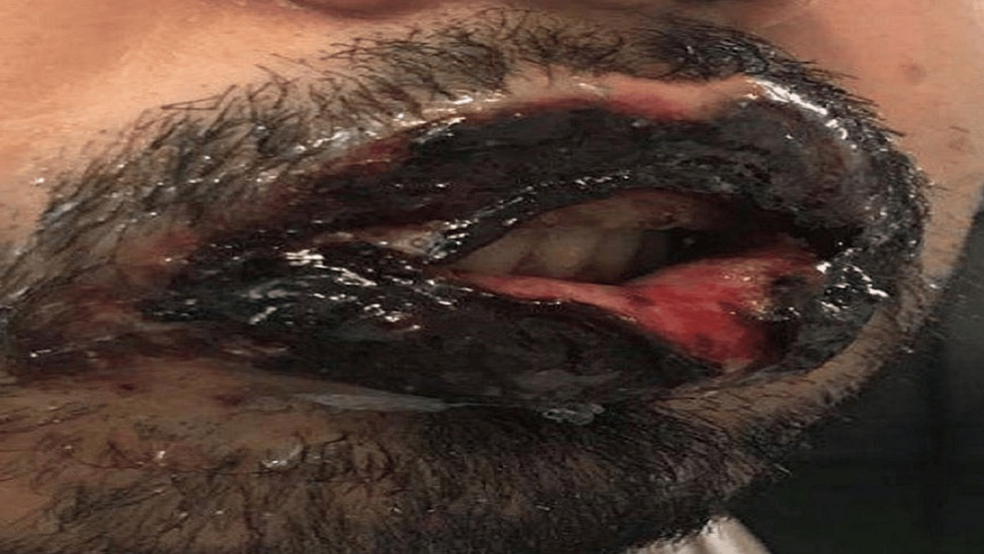
Oral mucosa and hemorrhagic crusting exudative lips.

**Figure 2:**
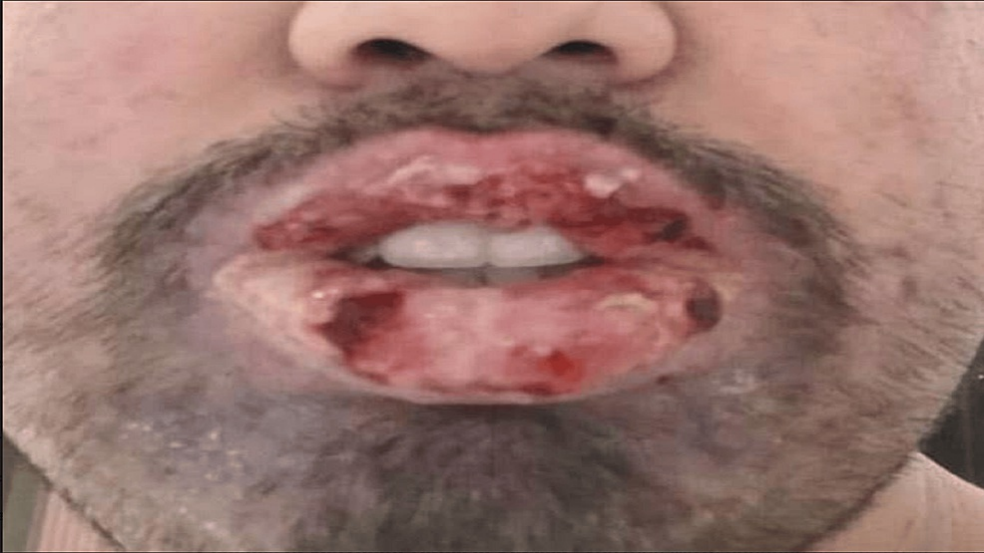
Oral mucosa and hemorrhagic crusting exudative lips.

Upon physical examination, the patient exhibited lesions affecting the conjunctiva, oral mucosa, and lips characterized by hemorrhagic crusting and exudation (Figures 1, 2). Additionally, polymorphic skin lesions were noted on the upper trunk, presenting as bullous erosions and ulcerations.

Following dermatology consultation, two punch biopsies were obtained for differential diagnosis, with specimens subjected to both light microscopy and direct immunofluorescence (DIF) analysis. The potential diagnoses considered included herpes zoster, PV, and bullous lichen planus or paraneoplastic pemphigus. Results from both diagnostic tests confirmed the presence of PV.

Light microscopy revealed features consistent with PV, showcasing suprabasal intraepidermal blistering alongside the presence of acantholytic keratinocytes, lymphocytes, and a sparse population of neutrophils and eosinophils within the blister space. Notably, the blister base exhibited a single layer of residual basal epidermal cells, with no significant lichenoid inflammatory infiltrate observed. Additionally, orthokeratosis of the stratum corneum was noted, while the deep dermis appeared unremarkable.

Routine blood and biochemical investigations, bone profile, and serology were done and the results are given in Table 1.

**Table 1:** Blood and biochemical investigations, bone profile, and serology. CMV: Cytomegalovirus, EBV: Epstein-Barr virus.

Investigation	Result	Reference range
White blood cell count	20.64	4.0 - 11.0 × 10^9/L
Neutrophils	14.3	40 - 60%
Hemoglobin	12.7	13.8 - 17.2 g/dL (men) / 12.1 - 15.1 g/dL (women)
Mean corpuscular volume (MCV)	85.1	80 - 100 fl
reticulocyte count	1.41	0.5 - 2.5%
Hematocrit	38.3	38.3 - 48.6% (men) / 35.5 - 44.9% (women)
platelet count	345	150 - 450 × 10^9/L
Sodium (Na)	135	135 - 145 mEq/L
Chloride (Cl)	105	96 - 106 mEq/L
Potassium (K)	4.2	3.5 - 5.0 mEq/L
Magnesium (Mg)	2.12	1.7 - 2.2 mEq/L
Uric acid	3.79	3.5 - 7.2 mEq/L
Blood urea nitrogen (BUN)	6.5	6 - 20 mEq/L
Calcium (Ca)	8.2	8.5 - 10.2 mEq/L
Alkaline phosphatase (ALP)	46	44 - 147 U/L
Procalcitonin	0.29	< 0.15 U/L
Aspartate aminotransferase (AST)	76	10 - 40 U/L
Alanine aminotransferase (ALT)	188	7 - 56 U/L
Total bilirubin	1.2	0.1 - 1.2 mg/dL
Direct bilirubin	0.4	0.0 - 0.3 mg/dL
Lactate dehydrogenase (LDH)	188	140 - 280 U/L
Haptoglobin	3.18	30 - 200 mg/L
Albumin	3.006	3.5 - 5.0 g/dL
Ferritin	345.14	30 - 400 mg/L (men) / 13 - 150 mg/L (women)
Herpes 1 IgG	Positive	Negative
CMV IgG	Positive	Negative
EBV IgG	Positive	Negative
Varicella zoster IgG	Positive	Negative

**Figure 3:**
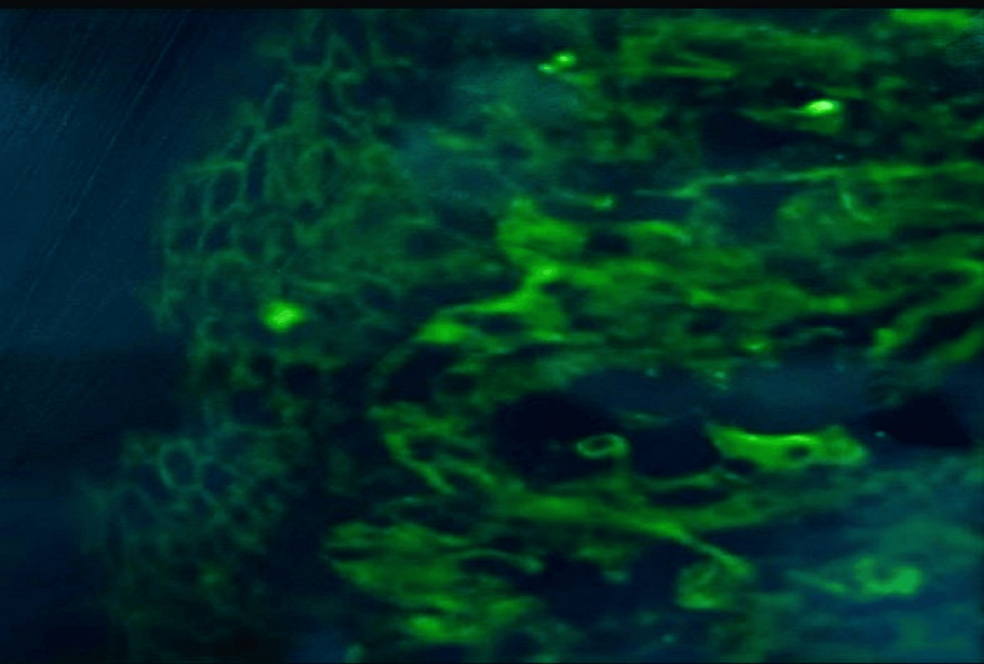
Direct immunofluorescence showed IgG ++ and c3 intraepidermal. ++ Moderate level of deposition.

Direct immunofluorescence findings further supported the diagnosis, demonstrating intense IgG and C3 deposition intraepidermally, forming a net-like pattern that was more pronounced in the lower epidermis. Conversely, no positive signals were detected in the subepidermal basement membrane zone. IgA, IgM, C1q, and fibrinogen showed either negative results or minor nonspecific signals (Table 2, Figure 3).

**Table 2:** Immunofluorescence (skin biopsy panel). ++ Moderate level of deposition.

Block	Antibody name	Result (s)	% Positive cells
T19-001936 B	IgA	-	N/A
T19-001936 B	IgG	++ Intradermal, net-like	N/A
T19-001936 B	IgM	-	N/A
T19-001936 B	C3	++ Intradermal, net-like	N/A
T19-001936 B	C1q	-	N/A
T19-001936 B	Fibrinogen	-	N/A

Additionally, a fluorescence in situ hybridization (FISH) panel was conducted, with results outlined in Table 3.

**Table 3:** Chronic lymphocytic leukemia (CLL) FISH panel. FISH: Fluorescence in situ hybridization.

Negative for deletion of ATM (11q22.3)
Negative for 12p13.2/ETV6 rearrangement
Negative for deletion of (13q14.3)
Negative for deletion of (17p13.1)

The patient initially received treatment with acyclovir for suspected herpes zoster infection, yielding no response. Subsequently, a skin biopsy was performed, confirming pemphigus as the primary skin lesion. Accordingly, steroid therapy commenced alongside weekly rituximab infusions at a dose of 375 mg/m2 for four weeks, albeit with limited response. Consequently, the patient was initiated on ibrutinib at a dosage of 420 mg once daily, resulting in a dramatic response in terms of mucocutaneous lesions. This treatment regimen persisted for two weeks, during which follow-up visits indicated noticeable improvement in the patient's condition.

## Discussion

PV is an acantholytic autoimmune disorder primarily affecting mucocutaneous membranes, characterized by the loss of cell-to-cell adhesion leading to the formation of bullae [6]. It is one of the three major types of pemphigus, alongside pemphigus foliaceus and paraneoplastic pemphigus [3]. While it impacts individuals across all ethnic groups, its highest prevalence occurs between the ages of 50 and 60, although the age of diagnosis varies significantly based on ethnicity and country of origin [6]. For instance, the median age of diagnosis in southern Saudi Arabia is reported to be 40 years [4].

The pathogenesis of PV entails the presence of immunoglobulin (Ig) antibodies targeting transmembrane glycoprotein desmogleins situated on the cell surface of keratinocytes. These desmogleins are integral components of desmosomes, crucial for cell adhesion. Consequently, the depletion of desmogleins leads to desmosomal remodeling, rendering cells susceptible to acantholysis [1]. Research indicates the involvement of B cells in the pathogenesis of PV [3].

The most prevalent lesion in PV is the blistering and erosion of oropharyngeal mucous membranes. Additionally, PV can affect other sites including the esophagus, genital system, anus, eyes, nose, and vocal cords. Systemic involvement is also observed in PV [7]. Two primary subtypes of PV exist, mucocutaneous and mucosal dominant. Both subtypes involve mucosal membranes, with the mucocutaneous type characterized by excessive cutaneous involvement. The Nikolsky sign is typically positive in PV [2].

PV is typically managed with corticosteroids and steroid-sparing agents such as mycophenolate mofetil and azathioprine [3]. Secondary infections are prevalent and can pose significant mortality risks, with respiratory tract infections, septicemia, and peptic ulcer disease being among the primary causes of death [8]. Histopathology and immunofluorescence serve as crucial tools in confirming the diagnosis of PV, with direct immunofluorescence emerging as the more reliable diagnostic test [3].

Ibrutinib, a BTK inhibitor, acts on an enzyme pivotal in the signaling pathways of numerous cells. BTK's functions extend to the development and maturation of B cells, as well as cytokine production, implicating it in autoimmunity and inflammation. Consequently, BTK inhibitors offer promise in the treatment of PV by modulating pathways associated with autoimmunity and inflammation [5]. As it is seen in our case the use of ibrutinib improved the symptoms of PV.

We present a case of PV in a 35-year-old patient concurrently diagnosed with CLL. Initially misdiagnosed as herpes zoster, treatment ensued but proved ineffective. Subsequent biopsy of the lesions definitively confirmed the diagnosis of PV. Notably, the patient exhibited asymptomatic CLL. Initiation of ibrutinib therapy led to significant amelioration of PV symptoms.

## Conclusions

Our case underscores the effectiveness of Bruton tyrosine kinase (BTK) inhibitors, particularly ibrutinib, in the management of pemphigus vulgaris (PV). Additionally, it highlights the potential for misdiagnosis of PV as other dermatological conditions, such as herpes zoster, necessitating thorough examination and diagnostic testing prior to treatment initiation. Following biopsy confirmation of PV, steroid therapy in conjunction with rituximab was initiated; however, the patient exhibited minimal improvement. Remarkably, the administration of ibrutinib precipitated the resolution of PV symptoms.
